# The effects of a carbonated beverage on the optical properties and microhardness of preheated bulk-fill composite resin restorations

**DOI:** 10.3389/froh.2025.1539527

**Published:** 2025-06-02

**Authors:** Nancy Soliman Farghal, Ayya Abu Shamleh, Osamah Al Hurmuzi, Okba Mahmoud

**Affiliations:** ^1^ Department of Restorative Dentistry and Endodontics, RAK College of Dental Sciences, RAK Medical and Health Sciences University, Ras Al-Khaimah, United Arab Emirates; ^2^ Department of Dental Biomaterials, Faculty of Dentistry, Tanta University, Tanta, Egypt; ^3^ Clinical Sciences Department, College of Dentistry, Ajman University, Ajman, United Arab Emirates; ^4^ Centre of Medical and Bioallied Health Sciences Research, Ajman University, Ajman, United Arab Emirates

**Keywords:** acidic drink, bulk-fill composite, color stability, carbonated beverage, giomer, gloss, microhardness, preheated composite

## Abstract

**Introduction:**

Preheating the high-viscosity forms of bulk-fill resin composites is recommended to enhance their flow and adaptability. Nevertheless, the impact of preheating on their characteristics upon exposure to carbonated beverages remains unclear. This study aims to evaluate the effect of a Cola beverage on the properties of preheated and non-preheated high-viscosity bulk-fill composite resins *in vitro*.

**Methods:**

Forty disc-shaped specimens were prepared from each of BEAUTIFIL-Bulk Restorative (BB) and Filtek One Bulk-fill (FOB) composite resin, then divided into two groups (*n* = 20), either preheated to 68°C for 15 min or kept at room temperature before polymerization, then specimens were immersed in Alkozay Cola beverage for 30 days (3 periods of 15 min/day). Color stability (ΔE_00_), surface gloss (GU) and Vickers Microhardness (VHN) were recorded before and after the Cola immersion. The data was analyzed with Two-way ANOVA, Three-way ANOVA and Tukey's HSD *post hoc* test using SPSS software at 95% significance level.

**Results:**

The color change was significantly higher in BB than FOB in all groups (*P* < 0:001), FOB had a significant reduction in color change after preheating (*P* < 0.05) while BB had no significant change (*P* > 0.05). Preheating significantly increased the gloss of BB and reduced that of FOB (*P* < 0.001), however, Cola beverage significantly reduced the gloss of all the groups (*P* < 0.001). Preheating significantly increased the microhardness of both materials (*P* < 0.001), however, Cola beverage significantly reduced the microhardness of all the groups (*P* < 0.001).

**Conclusion:**

Although the preheating of high-viscosity bulk-fill composites significantly improved their microhardness and improved the surface gloss of FOB, it did not protect both composites against the Cola drink attack. Preheated FOB showed improved color stability after the Cola immersion, but not to a clinically acceptable limit.

## 1 Introduction

Conventional resin-based composites have been successfully used in the field of aesthetic restorative dentistry for many years, however, their limited depth of cure into small increments of 2 mm thickness is still considered a critical point that limits their performance, rendering the conventional incremental composite placement technique sensitive and time-consuming (1, 2). Recently, a novel category of resin-based composites, known as “bulk-fill” composites, has been introduced to the dental market aiming at reducing the cost and saving time (3). The distinctive feature of these novel materials is their ability to be cured in a single step and placed in bulks of 4 mm thickness, in contrast to the conventional incremental placement technique, without affecting the degree of conversion, polymerization shrinkage, or their adaptation to the cavity walls and margins (4).

Bulk-fill resin composites are available in two different viscosities which highly influence their application efficiency and cavity wall adaptation. Low-viscosity bulk-fill resin composites are flowable in consistency which enables their adaptation to the cavity floors even in deeper and less accessible areas, this reduced viscosity is associated with decreased filler loading which reduces their surface wear resistance, and therefore, these low-viscosity forms must be capped with a layer of conventional composite materials. on the other hand, high-viscosity bulk-fill resin composites are designed to be used without a capping layer, as they have higher filler loading and enhanced mechanical properties, the higher filler loading and higher viscosity of the material reduce their adaptability to the cavity walls and margins and creates difficulty in sculpting the surface layer (5) (6). Accordingly, preheating the composite resin before its application is currently gaining popularity among dental practitioners due to its potential to enhance the extrusion and flow of high-viscosity composites, as well as for its potential to reduce microleakage and marginal adaptation (7). It has been demonstrated that the polymerization process is optimized and the degree of conversion is increased by increasing the temperature before the polymerization (8, 9).

In addition to enhancing the sculpting and handling characteristics of bulk-fill composite resins, it is anticipated that these direct restorative materials exhibit good aesthetic qualities and durability (10). The consumed oral beverages remain a critical point that affects the oral health (11). Despite advancements in the organic matrix and particle size of composite resins, color stability and gloss retention remain a prevalent issue for both patients and dentists (12, 13). The exposure of bulk-fill composite resins to different beverages in the oral environment may lead to color alteration due to intrinsic or external causes as reported in previous studies (14, 15). Besides, acidic beverages with low pH are also known to degrade the matrix structure of resins (16). Previous studies showed the negative effect of acidic beverages on the surface hardness of bulk-fill composite resin, which reflects the ability of the material to resist abrasion during function. Consequently, the restoration's durability may be influenced by the factors that impact its hardness (17–19).

Despite the reported advantages of preheating the composite resin materials on enhanced degree of conversion and improved marginal adaptation due to reduced viscosity (8, 20, 21) there is insufficient data about the preheating effect on optical characteristics and surface hardness of highly viscous bulk-fill composites. Thus, the current investigation aims to explore the influence of preheating two highly viscous bulk-fill composite resins on their color stability, surface gloss and microhardness after immersion in a commercial carbonated beverage. The first null hypothesis states that there will be no difference between the two materials regarding the tested properties. The second null hypothesis is that there will be no difference between the two materials in preheated and non-preheated state, and the third null hypothesis states that there will be no difference in the tested properties after immersion in the carbonated beverage.

## 2 Materials and methods

### 2.1 Study design

**Figure 1:**
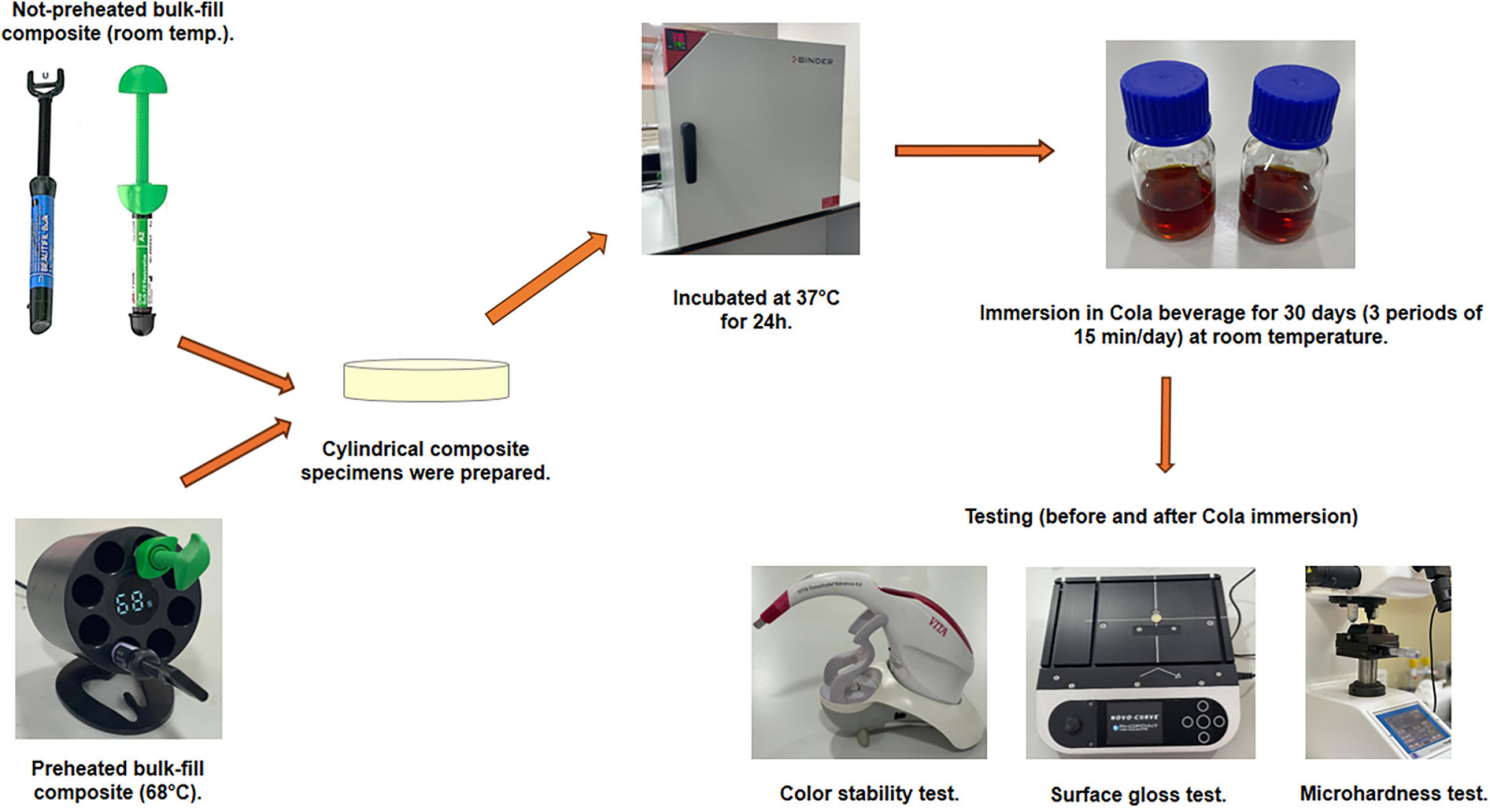
The experimental study design.

This study was conducted following the approval of the Research and Ethics Committee Ref. No: RAKMHSU-REC-9-2023/24-UG. Two high-viscosity bulk-fill composite materials were utilized in the current study, BEAUTIFIL-Bulk Restorative (BB), Shofu Inc, Kyoto, Japan, and Filtek^TM^ One Bulk-fill Restorative (FOB), 3M ESPE, St Paul, MN, USA. The details of each material are listed in (Table 1). Figure 1 illustrates the study's methodology.

**Table 1:** Material names, compositions and manufacturers of the study.

Brand name	Depth of cure, shade	Composition	Manufacturer	Lot no.
BEAUTIFIL-Bulk Restorative. (BB)	4 mm, A2	Matrix: Bis-GMA, UDMA, Bis-MPEPP, TEGDMA	Shofu Inc, Kyoto, Japan	022259
		Filler (w%/v%): 87/74.5 S-PRG filler based on fluoroboroaluminosilicate glass		
Filtek^TM^ One Bulk-fill. (FOB)	5 mm, A2	Matrix: AUDMA, AFM, UDMA, diurethane-DMA, and 1, 12-dodecane- DMA	3M ESPE, St Paul, MN, USA	9979066
		Filler (w%/v%): 76.5/58.4 ytterbium trifluoride, zirconia/Silica		

Abbreviations: AFM, additive fragmentation monomer; AUDMA, aromatic urethane dimethacrylate; DMA, dimethacrylate; Bis-GMA, bisphenol A glycidyl methacrylate; UDMA, urethane dimethacrylate; Bis-MPEPP, 2,2-bis(4-methacryloxy poly-methoxyphenyl) propane; TEGDMA, triethylene glycol dimethacrylate; wt%, weight percentage; v%, volume percentage.

### 2.2 Determination of the sample size

The sample size in the current study was calculated using the G*Power program v. 3.1.9.7 for Windows, based on four experimental groups. The power was set at 0.8, and the effect size was set to 0.4, as reported in previous studies (10, 22), with a 0.05 significance level. Accordingly, a minimum of 19 specimens per group was calculated and increased to 20 specimens per group to reduce sampling errors.

### 2.3 Fabrication of the specimens

A total of 80 disc-shaped specimens, 40 of each restorative material, were prepared. The specimens were fabricated utilizing a silicon mold (10 mm diameter & 2 mm thickness). The mold was positioned over a glass plate topped with a 10-mm wide polyester strip. After the application of each tested material within the mold, another similar polyester strip was delicately pressed with a glass plate over the specimen to eliminate excess material and attain a uniform smooth surface. Specimens were subjected to light curing for 20 s under the manufacturer's guidelines on top and bottom surfaces utilizing an LED curing light (Elipar™ DeepCure-L 3M ESPE, St Paul, USA) at an intensity of approximately 1,470 mW/cm^2^. The light intensity was frequently verified using the built-in radiometer to maintain a consistent light output. For the preheated specimens, a composite heater (AZDENT Dental Composite Heater, China) was used and its temperature was set at 68°C in which the composite syringes were kept for 15 min before use in each specimen fabrication. After polymerization, the specimens were incubated in distilled water at 37°C for 24 h to verify the completion of the polymerization process.

### 2.4 The grouping of the specimens

Specimens of each tested material were randomly divided into two groups (*n* = 20) according to their temperature before polymerization as follows:

• Not pre-heated/Positive control group (NH): Specimens kept at room temperature (25 ± 1°C) and polymerized without preheating.
• Preheated group (PH): Specimens were preheated to a temperature of (68 ± 1°C) before polymerization.

### 2.5 Carbonated beverage immersion of the specimens

The specimens were immersed in a carbonated Cola beverage, Alokozay Cola (Alkozay Cola Production Company, Kabul, Afganistan), pH 2.3, for 30 days of challenge, (3 periods of 15 min/day), at room temperature (25 ± 1°C), after each period of immersion, specimens were washed with distilled water, dried thoroughly and then re-immersed in the Cola beverage. Specimens were stored in an incubator after each immersion cycle in distilled water at 37°C. After 30 days, the specimens were finally washed in distilled water for 10 min and carefully dried before testing.

### 2.6 Testing of the specimens

#### 2.6.1 Color stability test

The color stability was tested by recording the difference in color parameters recorded for each specimen initially (baseline) and after immersion in the Cola beverage, utilizing a spectrophotometer (VITA Easyshade® Advance 4.0, Vita Zahnfabrik, Bad Säckingen, Germany) according to CIELAB color space system described by the Commission Internationale de l'Eclairage. Following calibration of the apparatus to correspond with the manufacturer's guidelines, the 5 mm-diameter spectrophotometer probe was positioned in the middle of the specimens; the specimens were positioned on a white non-reflecting surface to reduce interference from the background and under D65 Illumination source of light. Color changes (ΔE_00_) of each sample were calculated according to the CIEDE2000 equation as follows (23):

$$\Delta E_{00}=\left[{\left(\frac{\Delta L^{\prime }}{K_{L}S_{L}}\right)}^{2}+{\left(\frac{\Delta C^{\prime }}{K_{C}S_{C}}\right)}^{2}+{\left(\frac{\Delta H^{\prime }}{K_{H}S_{H}}\right)}^{2}+R_{T}\left(\frac{\Delta C^{\prime }}{K_{C}S_{C}}\right){\left(\frac{\Delta H^{\prime }}{K_{H}S_{H}}\right)}^{1/2}\right]$$

Where, ΔL΄, ΔC΄, and ΔH΄ color parameters correspond to the differences in Lightness, Chroma, and Hue, respectively. R_T_ is the rotation function, S_L_, S_C_, and S_H_ are weighting functions, K_L_, K_C_, and K_H_ are experiment correction parameters. In this investigation, these parametric variables were adjusted to 1 as a default value (13). The color stability values were further evaluated based on the 50:50% perceptibility (0.80) and acceptability (1.8) thresholds reported by Paravina, et al. (24), and according to the ISO/TR 28642:2016 (25).

#### 2.6.2 Surface gloss test

Gloss measurements were performed using Novo-Curve glossmeter (Rhopoint Instrumentation Ltd., UK) with a 60° light incidence and reflection angles following the International Organization for Standardization standard for medium gloss materials ISO 2813:2014 (26). Each specimen was placed over a 2 mm × 2 mm measuring window on the instrument, which was then covered with a black shield to prevent external light exposure during the measurement. The device was calibrated using a calibration plate supplied by the manufacturer before measuring each composite group. Gloss values were quantified in gloss units (GU). A completely non-reflective surface is ascribed to a value of zero (0 GU), whereas a highly polished surface with a refractive index of 1.567 reaches a value of 100 GU. For each specimen, three readings were recorded by rotating the specimen 120° angle and then averaged to obtain its gloss value as reported by Ardu, et al. (27) The gloss was evaluated before and after the Cola beverage immersion for all the specimen, the pre-immersion values of each specimen served as a negative control.

#### 2.6.3 Microhardness test

Microhardness was assessed with a Vickers microhardness tester (FM-800, Future-Tech Corp. Japan), employing a 136° pyramidal diamond indenter to create a square indent on each specimen. The indenter was applied to each specimen's surface with a test force of 100 g, sustained for a designated dwell duration of 15 s. The dimensions of the indent were ascertained visually by measuring the two diagonals of the square indent using a 40× objective lens. The mean of the two diagonals was employed to compute the Vickers Microhardness Number (VHN) utilizing the subsequent formula:

$$\mathrm{VHN}\;=\;1.854\;F/d^{2}$$

Where F: represents the applied force in Newton, and d: denotes the mean length of the two diagonals of each indentation. Three indentations for each specimen were documented and subsequently averaged to yield the final result. The microhardness was measured before and following the immersion in the Cola beverage, the pre-immersion values of each specimen served as a negative control.

### 2.7 Statistical analysis

Means and standard deviations were calculated for each group. Data was statistically analyzed using the Statistical Program for Social Sciences (SPSS®, version 27, IBM, NY, USA). The data was normally distributed after performing the Kolmogorov–Smirnov and Shapiro–Wilk test. Color stability was analyzed using Two-way Analysis Of Variance ANOVA, while surface gloss and microhardness were analyzed using Three-way ANOVA to evaluate the interactions among the various groups, then multiple pairwise comparison procedures were conducted using Tukey's HSD *post hoc* test at 95% level of significance (*p* < 0.05).

## 3 Results

### 3.1 Color stability results

**Figure 2:**
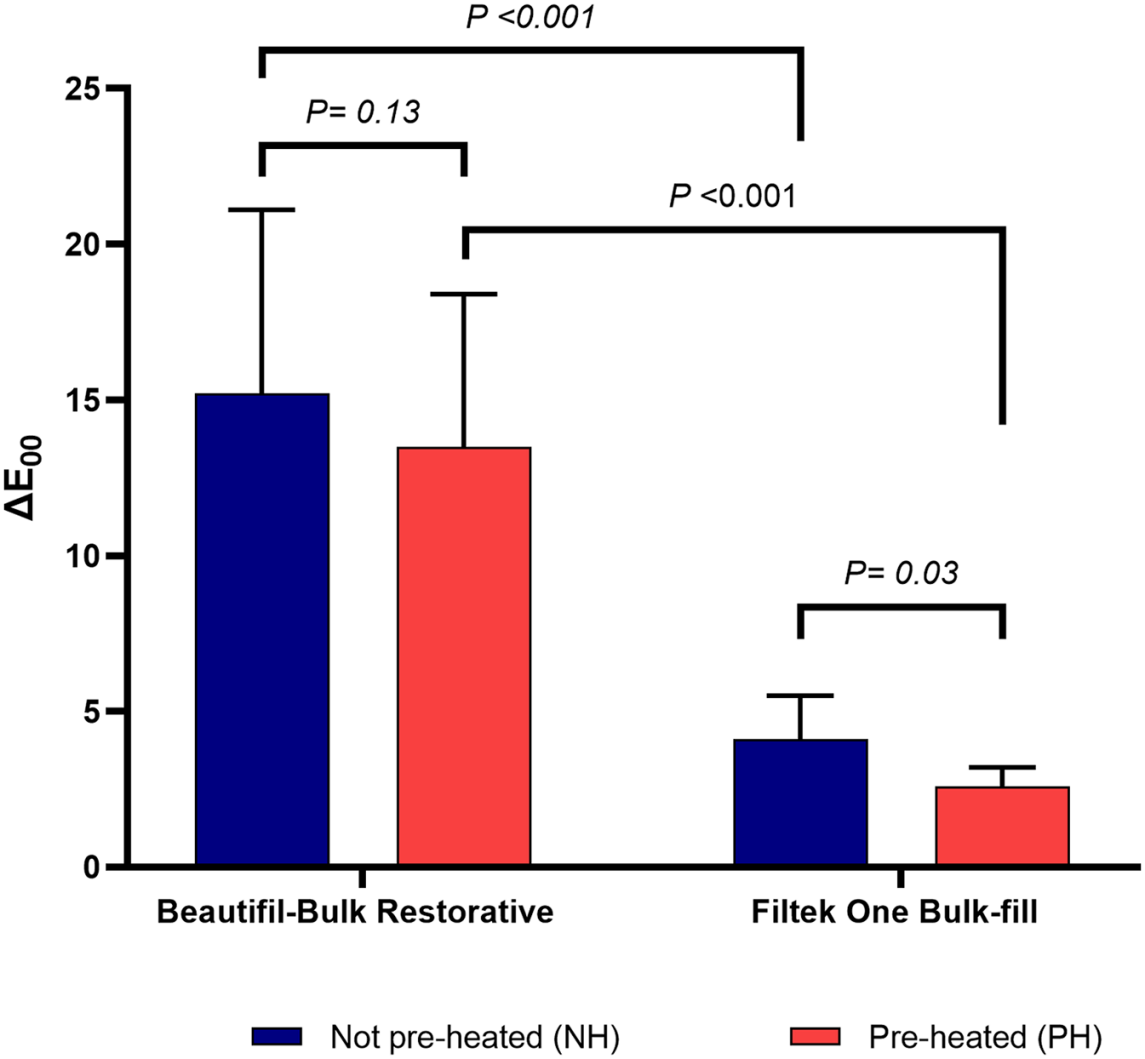
Bar chart showing the color stability (ΔE_00_) after Cola immersion of the tested materials, *P* ≤ 0.05 was considered significant.

The means and standard deviations of the color stability results are presented in Figure 2. Two-way ANOVA revealed significant interaction within and among the tested groups, *P* < 0.001 as shown in Table 2. BB showed no significant difference in color change between NH and PH groups; ΔE_00_ = 15.2 ± 5.9 and 13.5 ± (4.9) respectively, *P* = 0.13. Both NH and PH groups of BB color change values were significantly higher than the FOB groups, *P* < 0.001. The color change in FOB was significantly lower in the PH group than in the NH group 4.1 ± 1.4 and 2.6 ± 0.6 respectively, *P* = 0.03. The color change in all the groups of both tested composites was beyond the clinically acceptable ΔE_00_ threshold (>1.8).

**Table 2:** Two-way analysis of variance of the specimen's color stability after cola immersion (ΔE_00_) as affected by the composite type and the pre-polymerization temperature.

Source	Type III sum of squares	df	Mean square	F	*P*- value
Corrected model	2,280.564^a^	3	760.188	50.402	0.000*
Intercept	5,908.203	1	5,908.203	391.728	0.000*
Composite type	2,185.095	1	2,185.095	144.877	0.000*
Polymerization temperature	89.253	1	89.253	5.918	0.017*
Composite type × polymerization temperature	6.216	1	6.216	0.412	0.523
Error	1,146.263	76	15.082		
Total	9,335.030	80			
Corrected total	3,426.827	79			

^a^ R Squared = 0.666 (adjusted R Squared = 0.652).

* Significant at *P* ≤ 0.05.

### 3.2 Surface gloss results

Three-way ANOVA results revealed significant interaction within and among the tested groups, *P* < 0.001 as shown in Table 3. Means and standard deviations of the surface gloss results are presented in Table 4. Initially, the FOB-NH group showed significantly higher gloss values (125.8 ± 22.3) compared to the BB-NH group (58.3 ± 11.6), *P* < 0.001. However, the BB-PH groups had a significantly higher gloss value (112.3 ± 19.4) compared to the FOB-PH group (64.5 ± 6.3), *P* < 0.001. After immersion in the Cola beverage, all the groups showed a significant reduction in gloss compared to their initial readings, *P* < 0.001, the highest values were recorded for FOB-PH (34.8 ± 11.6) and BB-NH (34.4 ± 12) with no significant difference between both, *P* = 0.94, followed by FOB-NH (22.8 ± 3.7) and BB-PH (23.4 ± 16.2) with no significant difference between both as well, *P* = 0.89.

**Table 3:** Three-way analysis of variance of the specimen's surface gloss (GU) as affected by the composite type, the pre-polymerization temperature and the immersion in cola.

Source	Type III sum of squares	Df	Mean square	F	*P*- value
Corrected model	221,920.094^a^	7	31,702.871	158.318	0.000*
Intercept	567,440.041	1	567,440.041	2,833.687	0.000*
Composite type	941.870	1	941.870	4.704	0.032*
Polymerization temperature	101.761	1	101.761	0.508	0.477
Cola immersion	150,638.802	1	150,638.802	752.261	0.000*
Composite type × polymerization temperature	21,330.542	1	21,330.542	106.521	0.000*
Composite type × cola immersion	988.036	1	988.036	4.934	0.028*
Polymerization temperature × cola immersion	170.982	1	170.982	0.854	0.357
Composite type x polymerization temperature × cola immersion	47,748.100	1	47,748.100	238.445	0.000*
Error	30,437.685	152	200.248		
Total	819,797.820	160			
Corrected total	252,357.779	159			

^a^ R Squared = 0.879 (adjusted R Squared = 0.874).

* Significant at *P* ≤ 0.05.

**Table 4:** The surface gloss (GU) means and standard deviations of the materials utilized in the current study.

Materials/temperature	Not pre-heated (NH)		Pre-heated (PH)	
Beautifil-bulk restorative (BB)	Before immersion	After immersion	Before immersion	After immersion
	58.3 ± (11.6)^Aa^	34.4 ± (12)^Ab^	112.3 ± (19.4)^Ac^	23.4 ± (16.2)^Ad^
Filtek one bulk-fill (FOB)	125.8 ± (22.3)^Ba^	22.8 ± (3.7)^Bb^	64.5 ± (6.3)^Bc^	34.8 ± (11.6)^Bd^

Different letters within columns and lines indicate statistically significant differences (*p* < 0.05). Lowercases represent linear differences while uppercases represent columnar differences.

### 3.3 Microhardness results

Three-way ANOVA revealed significant interaction within and among the tested groups (*P* < 0.001) as shown in Table 5. Means and standard deviations of the microhardness results are presented in Table 6. Comparing the effect of pre-polymerization temperature on BB: initially; the microhardness was significantly higher in PH group (53.2 ± 9.7) than NH group (40.1 ± 7.3), *P* = 0.001. The microhardness of the same material significantly reduced after immersion in the acidic beverage in both groups compared to their initial readings *P* < 0.001, to be (30.9 ± 8.5) for BB-NH group which was not significantly different from BB-PH group (26.1 ± 13.3), *P* = 0.23. The microhardness of FOB was significantly higher in all the groups than BB, *P* < 0.001. Comparing the effect of pre-polymerization temperature on FOB: initially the microhardness of FOB-PH group (68.5 ± 10.4) was significantly higher than NH group (51.8 ± 4.5), *P* = 0.001, however after immersion in the acidic beverage, both groups showed significant reduction in microhardness, *P* < 0.001, to be (44.9 ± 6.1) for FOB-PH, and (42.9 ± 8.3) for FOB-NH, with no significant difference between both groups, *P* = 0.63.

**Table 5:** Three-way analysis of variance of the specimen's Vickers microhardness (VHN) as affected by the composite type, the pre-polymerization temperature and the immersion in cola.

Source	Type III sum of squares	Df	Mean square	F	*P*- value
Corrected model	24,957.296^a^	7	3,565.328	47.732	0.000*
Intercept	320,945.434	1	320,945.434	4,296.770	0.000*
Composite type	8,340.255	1	8,340.255	111.658	0.000*
Polymerization temperature	1,822.635	1	1,822.635	24.401	0.000*
Cola immersion	11,798.882	1	11,798.882	157.962	0.000*
Composite type × polymerization temperature	265.277	1	265.277	3.551	0.061
Composite type × cola immersion	36.119	1	36.119	0.484	0.488
Polymerization temperature × Cola immersion	2,670.119	1	2,670.119	35.747	0.000*
Composite type × polymerization temperature × cola immersion	24.010	1	24.010	0.321	0.572
Error	11,353.575	152	74.695		
Total	357,256.305	160			
Corrected total	36,310.871	159			

^a^ R Squared = 0.687 (adjusted R Squared = 0.673).

* Significant at *P* ≤ 0.05.

**Table 6:** The vickers microhardness (VHN) means and standard deviations of the materials utilized in the current study.

Materials/temperature	Not pre-heated (NH)		Pre-heated (PH)	
Beautifil bulk (BB)	Before immersion	After immersion	Before immersion	After immersion
	40.1 ± (7.3)^Aa^	30.9 ± (8.5)^Ab^	53.2 ± (9.7)^Ac^	26.1 ± (13.3)^Ab^
Filtek one bulk fill (FOB)	51.8 ± (4.5)^Ba^	42.9 ± (8.3)^Bb^	68.5 ± (10.4)^Bc^	44.9 ± (6.1)^Bb^

Different letters within columns and lines indicate statistically significant differences (*p* < 0.05). Lowercases represent linear differences while uppercases represent columnar differences.

## 4 Discussion

The current *in vitro* study investigated the effect of a carbonated Cola beverage on the color stability, surface gloss and microhardness of two bulk-fill composite resins in preheated and non-preheated conditions before their polymerization. The first and third null hypotheses were rejected due to significant differences in the tested properties between the two resin composites and the impact of the carbonated beverage on all properties. The second null hypothesis was partially rejected, as preheating had no significant effect on the color change of BB.

The two investigated bulk-fill restorative materials in the current study were selected as they have different formulations both in their organic resin matrix and inorganic filler, they can be used in anterior and posterior restorations according to their manufacturer's claims. Beautifil-Bulk restorative is based on surface pre-reacted glass (S-PRG) fillers technology which imparts sustained fluoride release with an anti-plaque effect, this makes it beneficial for cases with high caries risk due to its ability to minimize secondary caries formation. Filtek One bulk-fill contains innovative monomers that reduce shrinkage and shrinkage stress, they also possess nano-ﬁller technology which provides superior aesthetic properties. Both composites also have different depth of cure levels, 4 and 5 mm respectively, thus they have a wide range of applications among dental practitioners.

Preheating of the bulk-fill composites was compared to room temperature polymerization in the current study since it became a popular technique and gained attention lately. The flow of composite polymers can be enhanced by increasing their temperature, as demonstrated by recent literature (7). Some of the potential advantages of composite preheating include enhanced marginal adaptation, better handling, and a higher degree of monomer conversion. A previous study by Kampanas (28) found that the average preheating temperature of bulk-fill composites which was considered safe and not harmful to the pulp tissues was between the range of 54°C and 68°C, thus, the preheating temperature in the current investigation was set to 68°C to maximize the benefits of the preheating technique.

Another critical factor to consider when preheating is the sufficient time to achieve optimal flow and enhance the properties of the restorative material. Previous studies that referenced the required time for material heating revealed a wide range of minimum and maximum times. Nevertheless, a clinical time of approximately 15 min is considered reasonable, as per the findings of Mohammadi, et al., and Karacan and Ozyurt (29, 30). Accordingly, 15 min of preheating time was considered in the current investigation.

It is crucial to ascertain whether the behaviour of the investigated materials has clinical implications and to consider its clinical acceptability during function. Therefore, the color stability results in the current study were statistically analyzed to assess the significant differences between groups, besides, it was further assessed based on the 50:50% acceptability threshold (AT) and the perceptibility threshold (PT) in CIEDE2000 which were 0.8 and 1.8, respectively, as reported by Paravina, et al. (31) and as aligned with ISO standards (25). The susceptibility of the composite resins to staining may be related to their hydrophilicity, the degree of conversion, as well as to the water sorption of the resin matrix (16, 32). Although the color change of both tested materials was beyond the 50/50% acceptability limit (>1.8), the color change of BB composite was significantly higher than FOB in both preheated in non-preheated states, this may be related to the difference in resin matrix composition between both materials. While both composites contain UDMA, which may reduce hydrophilicity and water absorption thus enhancing the color stability of composite resins, BB contains the more hydrophilic monomers Bis-GMA and TEGDMA, which are reported to increase the discoloration of composites by Ren, et al. (33), and Ozera, et al. (16). Moreover, BB is a bulk-fill form of giomer composites, representing a distinct category of bioactive resin-based materials capable of releasing fluoride as a preventive mechanism. However, the fluoride-release process may potentially create voids within the resin matrix, which in turn can reduce color stability due to enhanced pigment retention (34). Consequently, a more significant color shift with the exposure to the Cola beverage occurred in BB composite. The presence of these hydrophilic monomers and the bioactive characteristics also explain the current finding that preheating of composites was not able to stabilize the color of BB, this finding is in line with the study by Daneshpooy, et al, who found a significant color change of giomer composite after preheating in tea solution compared to microhybrid and nanohybrid composites (35). On the other hand, preheating of FOB in the current study improved it's the color stability after the Cola beverage attack, this is probably due to the presence of the unique additive fragmentation monomer (AFM), which increases the formation of cross-links between adjacent polymer chains, the preheating seems to improve the reactivity of this monomer and increase the degree of conversion which in turn improves the color stability by reducing the free monomer chains exposed to the colorant solution as reported in studies by Sousa, et al. (36) and Darabi, et al. (37). The current study result, however, is not in line with a study by Abdulmajeed, et al. (22) who reported in their study that preheating had no effect on stabilizing the color of the FOB composite, probably due to the strong staining potential of the coffee utilized in their study (13).

Surface gloss is an optical feature that is determined by the intensity of light reflection. The angle of incident light, the refractive index of the material components, and surface features are several elements that influence gloss (38). This study employed a 60° angle of incident light as per ISO 2813:2014 standards for medium gloss materials (26), rendering the gloss values contingent upon the surface topography and the material's refractive index. Irregular surface topography disperses light rather than reflecting it, hence diminishing the gloss value (39). There is no definitive threshold for gloss values of dental composites; however, it is advised to maintain a gloss value within the 40–60 GU range (40).

A recently published systematic review concluded that surface gloss is a reliable indicator for evaluating the effectiveness of finishing and polishing procedures on composite resin surfaces, as it directly correlates with surface roughness (41). Moreover, previous studies have demonstrated that the smoothest composite surfaces are obtained when polymerized against Mylar strips, without undergoing mechanical finishing (42, 43). In line with this evidence, the specimens in the current study were polymerized against polyester celluloid strips without additional finishing or polishing. This approach was intended to eliminate operator-dependent variability associated with polishing procedures, ensuring that gloss measurements reflect the intrinsic surface characteristics of the composite materials in their preheated and non-preheated states, thereby revealing their baseline performance without alterations induced by polishing. In the current study, both materials exhibited satisfactory gloss values before the immersion in Cola beverage, with FOB showing superior gloss compared to BB, this may be attributed to the difference in filler load as well as the filler size and distribution in both materials, FOB filler is composed of agglomerated and non-agglomerated nanosized filler with total volume of 58.4% which is less than the 74.5% SPG-R filler of BB. It was reported in previous studies by Alhassan, et al., and (44) that composites having a filler size between 5 and 20 nm are associated with an increase in gloss and light reflection, as these fillers are smaller than the visible light 400–800 nm wavelength. The high filler loading is also associated with less degree of conversion as suggested by Dionysopoulos, et al. (45) and Bucuta and Ilie (46), and this explains the improved gloss values of BB composites after preheating, which can be attributed to the improvement in the degree of conversion of the resin matrix secondary to its preheating. Although preheating reduced the gloss values of FOB, its gloss values remained clinically acceptable, this reduction may be related to stress build-up within the resin matrix secondary to the fastened polymerization rate after preheating as reported in Deb, et al. (21), and El-Korashy (47) studies, which may alter the bonding between the filler particles and resin matrix, thus changing the light reflection and subsequently reducing the surface gloss. On the Other hand, the gloss values reduced dramatically to be clinically unacceptable in all groups upon Cola exposure, which is in line with previous studies by Zovko, et al. (48) and Ozera, et al. (16). Such decrease in gloss is thought to be due to the presence of phosphoric acid in the composition of the Cola drink, which softens the organic matrix and thus leads to a change in light refraction and consequently a decrease in surface gloss.

The microhardness values in the current study was generally material dependent, the higher VHN recorded for FOB in all groups compared to BB is more likely related to the presence of the nano-sized filler particles which reduces the interstitial spaces, thus improving the surface hardness as reported in a study by Yap, et al. (49). The preheating showed a significant increase in microhardness of both tested composite resins in the current investigation compared to non-preheated groups without the acidic challenge, this finding is in line with previous studies (50–53). The degree of conversion of carbon double bonds in the composite resin matrix is often reflected in its hardness values, thus increased hardness following preheating is highly related to the improvement in the degree of conversion and therefore greater cross-linking in the monomer chains (54). According to Trujillo, et al. (55), and Daronch, et al. (56) the rate of monomer-to-polymer conversion can be enhanced by warming a composite resin at biologically compatible conditions. However, a significant reduction in microhardness values occurred in both materials after exposure to the Cola drink in the preheated and non-preheated state, the deterioration effect of acidic drinks on composites microhardness is in line with Borges, et al. (17), and Poggio, et al. (57), studies. This is more likely due to softening of the organic matrix by the action of phosphoric acid on of both composites, hence changing the bonding between the silane coupling agent and filler particles, however, the reduction in hardness was less in FOB, probably due to its harder zirconia filler content compared to the surface pre-reacted glass filler (SPG-R) in BB restorative composite. Besides, the inclusion of nanoclusters alongside nanoparticles in FOB filler diminishes the interstitial space, hence enhancing the physical properties and the surface hardness.

The findings of the current study are limited to the characteristics of *in vitro* studies, some oral environmental conditions were not included, such as the change in intraoral temperature, the effect of other beverages and the diluent effect of saliva. Future research should explore a broader range of preheating temperatures and investigate the effects of different beverages on composite materials. Additionally, further studies are recommended to assess other clinically relevant outcomes such as mechanical properties, and the long-term durability of preheated bulk-fill composites, particularly after thermal cycling and under clinical conditions.

## 5 Conclusions

Given the constraints of the present studies, it can be concluded that preheating improved the color stability of FOB and the gloss of BB, it also improved the microhardness of both bulk-fill composites. Conversely, exposure to Cola adversely affected all properties, resulting in clinically unacceptable color alterations.

## Data Availability

The raw data supporting the conclusions of this article will be made available by the authors, without undue reservation.
